# A Simple and a Reliable Method to Quantify Antioxidant Activity In Vivo

**DOI:** 10.3390/antiox8050142

**Published:** 2019-05-22

**Authors:** María Pilar de Torre, Rita Yolanda Cavero, María Isabel Calvo, José Luis Vizmanos

**Affiliations:** 1 Department of Pharmaceutical Technology and Chemistry, School of Pharmacy and Nutrition, University of Navarra, Irunlarrea 1, 31008 Pamplona, Spain; mdetorre@alumni.unav.es; 2 Department of Environmental Biology, School of Sciences, University of Navarra, Irunlarrea 1, 31008 Pamplona, Spain; rcavero@unav.es; 3 IDISNA-Instituto de Investigación Biosanitaria de Navarra, 31008 Pamplona, Spain; jlvizmanos@unav.es; 4 Department of Biochemistry & Genetics, School of Sciences, University of Navarra, Irunlarrea 1, 31008 Pamplona, Spain

**Keywords:** *C. elegans*, DPPH scavenging, green tea, rosmarinic acid

## Abstract

The characterization of compounds with antioxidant activity is of great interest due to their ability to reduce reactive oxygen species production and, therefore, prevent some age-related diseases. Its antioxidant capacity can be analyzed by different methods both in vitro and in vivo. *Caenorhabditis elegans* is an in vivo model widely used in ageing research. Until now, available tests analyze functional effects in the worms, so the antioxidant activity of the compound is indirectly monitored. We have developed a simple and a reliable method to quantify internal antioxidant activity in vivo. To validate this method, we analyzed an aqueous green tea extract and two other compounds with a well-known antioxidant activity and without this activity. The results obtained (EC_50_ green tea = 21.76 ± 1.28 µg/mL; EC_50_ positive control = 8.50 ± 0.33 µg/mL; negative control EC_50_ > 500 µg/mL) can help in the design of further in vivo experiments. Thus, our method can be used as a previous screening capable of reducing the gap between in vitro and in vivo assays.

## 1. Introduction

Living organisms are very complex systems in which a plethora of enzymatic reactions that require oxygen take place. Thus, molecular oxygen (O_2_) is a main component of the metabolism and the production of energy but it can also be present as very reactive short-lived derivatives (ROS, reactive oxygen species) such as superoxide (O_2_•^−^), hydrogen peroxide (H_2_O_2_), and the hydroxyl radical (•OH). These molecules can cause cell damage, the most important ones affecting DNA and polyunsaturated fatty acids in the membrane [1]. To counteract such damages, organisms have developed several antioxidant defence systems. However, according to the ageing and the free radical theory [2] the efficiency of the these protective systems decrease with age, and the accumulation of these species can favor the development of some diseases such as Alzheimer’s [3] and diabetes [4].

One of the most important areas of current research in nutrition lay in the search for compounds that are capable of reducing the production or the reactivity of ROS. Besides, there is a general awareness for the quality and the sustainability in the nutritional products. All of this is causing both industry and basic research to focus on the search of natural and traditional products as supplements with a high antioxidant activity [5,6].

The antioxidant activity can be analyzed by different methods both in vitro and in vivo. Among in vitro assays, the DPPH•-based method is probably the most popular one due to its simplicity, speed, and low cost [7]. DPPH• (1,1-diphenyl-2-picrylhydrazyl) is a stable free radical that can be reduced by transferring a hydrogen from other compounds. On the other hand, in vivo assays allow an analysis under physiological conditions but require the use of animal models, some of them (like mammals) are expensive and very time-consuming. By contrast, *C. elegans* is a simpler and economical model that makes use of the deep knowledge acquired about its biology in the last years and the conservation with mammals of some of the metabolic pathways. Different knock-out and transgenic strains are available that allow the monitoring of different enzymatic activities. For example, some antioxidant detoxifying enzymes include superoxide dismutase (SOD), catalase (CAT), and gluthatione peroxidase (GPx) that can be monitored as fluorescently tagged proteins in this organism [8]. Additionally, its short lifespan has allowed the development of tests to quantify stress resistance against abiotic factors like oxidation (Paraquat, Juglone), hypoxia, and temperature [9]. Antioxidant activity can be inferred from a combination of these assays comparing the results from treated and non-treated worms. Therefore, while in vitro methods quantify the antioxidant activity of a compound directly, in vivo methods do so indirectly for example by quantifying an overexpression of a fluorescent tagged protein or by an increase in the lifespan. However, there are some drawbacks in these approaches. When we focus on an enzymatic activity, we must consider that the effect may be due to another non-tested protein. When quantifying a functional effect, we must consider that it may be the result of additional activities of this compound in this model. Besides, these functional assays are carried out simultaneously with others that quantify pharyngeal pumping to ensure that worms are eating, since a caloric restriction affects lifespan [10]. In some cases, it must also be demonstrated that the compound is ingested, for example by tracing it with fluorescence (although in most natural compounds this tracing is not possible). Thus, in most cases it is assumed that the worms eat the compound because a functional effect can be observed, and this compound is attributed an in vivo antioxidant activity because the in vitro activity had been previously demonstrated. 

To fill the gap existing between in vitro and in vivo methods, we have developed a method that is capable to directly quantifying antioxidant activity inside *C. elegans*. To test this method, we selected a compound, green tea extract, with a well-known antioxidant activity. Green tea (*Camellia sinensis* (L.) Kuntze, *Theaceae*) infusion is one of the most common beverages in the world whose consumption is increasing and has a wide range of properties like anti-inflammatory, antioxidant, fatigue relief, and cancer prevention [11,12,13]. When compared to other teas, green tea has the highest concentration of polyphenols, such as gallic acid and epigallocatechin (EGC) [12]. These polyphenols have well-known antioxidant activity. In fact, as a positive control for a high antioxidant activity, we chose rosmarinic acid [14] that is a natural phenol carboxylic acid synthetized by many medicinal herbs [15]. By contrast, glucose was analyzed as a negative control. This monosaccharide is also present in medicinal plants, but lacks antioxidant activity. Results obtained with this method agree with these data.

## 2. Material and Methods

### 2.1. Preliminary Assay

On a preliminary determination with the microscope, a slightly color change inside worms which had received a treatment of rosmarinic acid was detected when a DPPH• solution was added to the medium. This fact pointed to rosmarinic acid actually being inside the worm but also that the antioxidant activity of this compound (or a derivative) was preserved there. For this reason, we decided to quantify this activity.

### 2.2. Green Tea Extract Preparation

Aqueous green tea extract was prepared from commercial dried leaves by using the same procedure as on previous studies at 4 °C [16]. Finally, we obtained a lyophilized material (lyophilization at −45 °C and under 0.6 mbar—dry leaves-extract ratio 3:1) to ensure its stability and facilitate subsequent dosage.

### 2.3. Antioxidant Activity In Vitro

Antioxidant activity can be monitored using the scavenging effect of radicals on DPPH• (cat. no. D9132, Sigma-Aldrich Co., St. Louis, MO, USA) [17]. We tested green tea at 10 different concentrations (1000, 500, 250, 125, 62.5, 31.25, 15.63, 7.81, 3.91, and 1.95 µg/mL) diluting the previous solution by half. In addition, same concentrations were tested for rosmarinic acid (cat. no. 536954, Sigma–Aldrich Co., St. Louis, MO, USA) and glucose (cat. no. G5767, Sigma–Aldrich Co., St. Louis, MO, USA) as positive and negative controls, respectively. To do that, 150 µL of the sample was mixed with 150 µL of DPPH• 0.04 mg/mL (cat. no. G5767-25G, Sigma-Aldrich Co., St. Louis, MO, USA). The reaction was monitored every 15 min up to 90 min. Absorbance at 517 nm (spectrophotometer UV PowerWave XS, BioTek Instruments, Inc., Winooski, VT, USA) was used to calculate radical scavenging activity (% of inhibition) with the formula

$$Inhibition\;\left(\%\right)=1-\frac{Abs_{sample}-Abs_{blank}}{Abs_{control}-Abs_{blank}}\times 100$$

where *Abs_sample_* was the absorbance of the reaction in presence of sample (sample dilution+DPPH solution), *Abs_blank_* was the absorbance of the blank for each sample dilution (sample dilution+DPPH solvent) and *Abs_control_* was the absorbance of control reaction (sample solvent+DPPH solution).

Then, this value obtained for every concentration was plotted to obtain EC_50_ values (concentration in which the 50% of the free radical DPPH is reduced) in each time point.

### 2.4. Antioxidant Activity In Vivo

#### 2.4.1. Worms and Plate Preparation

*C. elegans* was cultured as described previously [18]. The strain used was N2 *Bristol* as wild-type reference strain provided by the *Caenorhabditis Genetics Center* (CGC, University of Minnesota, Minneapolis, MN, USA). All assays were performed in 6-well cell culture plates with 4 mL of Nematode Growth Medium (NGM) per well at 20 °C (FOC 215i Cooled Incubator, VELP Scientifica, Usmate, Italy). We tested five different concentrations of green tea, rosmarinic acid (RA) as positive control and the glucose as negative control of antioxidant activity (40 µL of each per well: 50, 20, 10, 1, 0.1, and 0 mg/mL in sterile water). 100 µL of *Escherichia coli* OP50 were seeded on each well as a worm food source. 

**Figure 1:**
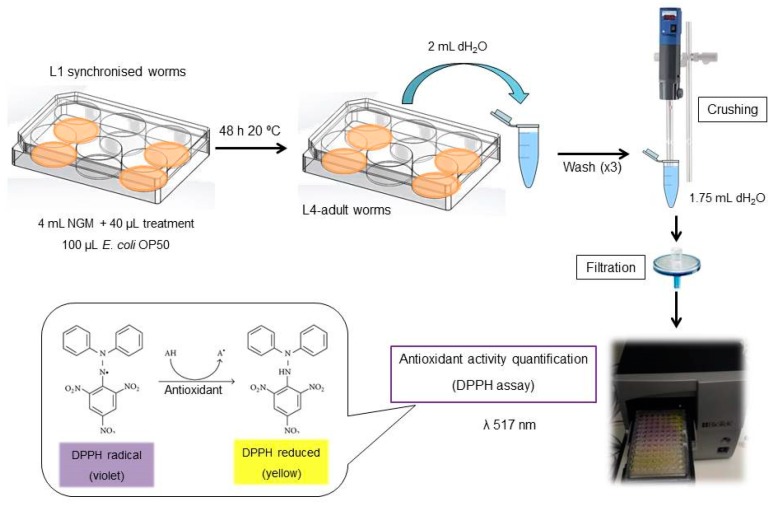
Step-by-step protocol for the proposed method.

2000 L1 synchronized worms [19] were placed onto each well and collected 48 h later, washing with 2 mL of sterile water per plate. After washing and a brief centrifugation at 314 g 4 min 20 °C, worms were resuspended in 1.75 mL of sterile water and crushed using Ultraturrax T25 (IKA-Werke GmbH & Co. KG, Staufen, Germany) for 20 s at maximum power. Solutions were then filtered to separate cellular debris using a 0.45 mm filter (cat. no. SLGP033RS, Sigma-Aldrich Co., St. Louis, MO, USA) to avoid interferences in the UV-spectrometry (Figure 1).

#### 2.4.2. DPPH• Assay

Antioxidant activity was quantified with DPPH• following the procedure explained before. In this case, the number of worms was the same in each condition, so aliquots of 150 µL of each filtered worm solution were added to a 96-well plate and made react with 150 µL of DPPH• 0.04 mg/mL. The reaction was monitored as before, obtaining the corresponding EC_50_ value for each time point.

#### 2.4.3. Limitations and Precautions of the Method

Worms were exposed to treatment during its growth from L1 to L4-adult at 20 °C (48 h); these are the same conditions that are applied in functional assays such as survival and gene expression. A change in any of these conditions or the number of worms placed onto the plates (2000 per well) may change the values obtained. Although the initial number of worms is controlled, there might be little experimental losses during the process. However, in this case, it seems that results are not affected, as data is not spread. Alternatively, data could be normalized using protein quantification. Since a wide concentration range of the compound is needed in order to calculate the EC_50_ value, it is very important that both the conditions and the number of worms used remain constant. In addition, non-treated worms (0 mg/mL of compound in the same solvent) need to be monitored in parallel. For the 96-well plate absorbance quantification, control (DPPH• + solvent), blank (solvent + methanol), and sample blank (sample + methanol) are needed according to DPPH• assay in vitro 96-well plate design [17]. Methanol is used because DPPH• is diluted in this solvent.

### 2.5. Statistical Analysis

DPPH• quantification was replicated three times for each condition for both the in vitro and the in vivo assays. EC_50_ values were generated with GraphPad Prism v6.01 (GraphPad Software, La Jolla, CA, USA). Average, standard deviation calculation and graphs were performed with Microsoft Excel (2010, Microsoft Corp., Redmond, WA, USA). Statistical analysis was performed using Stata v.12 (StataCorp LLC, College Station, TX, USA). Normality was checked by Shapiro-Wilk test. Statistical differences were estimated by ANalysis Of Variance (ANOVA) followed by pairwise comparison post hoc test using Tukey’s method (95% confidence level, *p* < 0.05).

## 3. Results

**Figure 2:**
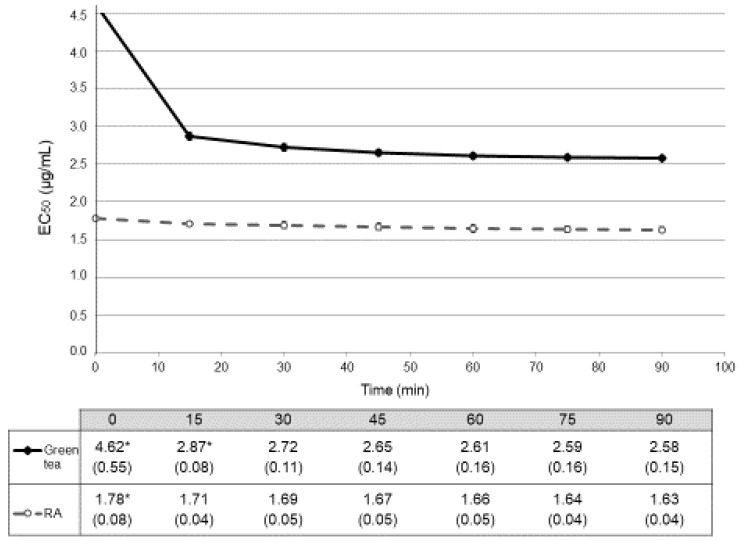
Temporal evolution of antioxidant activity in vitro (DPPH•) expressed in EC_50_ (mean ± SD µg/mL). Dark spots and continuous line correspond to data from treatment with green tea. Empty spots and dashed line correspond to data from treatment with rosmarinic acid (RA). Antioxidant activities of both green tea and RA showed differences in all the time-points (*p* < 0.001). Table below the graph shows the EC_50_ mean values (SD) µg/mL. * indicates the time points in which statistical differences (*p* < 0.05) with their consecutive value could be observed.

In the in vitro assay, the reaction with DPPH• method was monitored at 517 nm every 15 min for 90 min. Radical scavenging activity (% inhibition) calculated for each concentration at each point were collected (Table 1) and expressed as EC_50_ values and represented as a function of time (Figure 2). Glucose did not reach 50% of scavenging activity, being the EC_50_ values higher than 1000 µg/mL. 

We considered that chemical reaction was stable when no statistical differences (*p* > 0.05) could be observed between two consecutive values. For the green tea, reaction stabilized after 30 min (EC_50_ = 2.72 ± 0.11 µg/mL; with a *p* = 0.120 between EC_50_ at 30 min and EC_50_ at 45 min) (Figure 2). For this reason, we considered that this value can be representative for the antioxidant activity of green tea in vitro. In the case of RA, antioxidant activity stabilized early, with an EC_50_ at 15 min 1.71 ± 0.04 µg/mL (and a *p* = 0.479 between values at 15 min and at 30 min) so we chose this value as the representative one for the antioxidant activity of this compound. Antioxidant activities in vitro of both green tea and RA were also different at all time-points (*p* < 0.001) (Figure 2).

**Figure 3:**
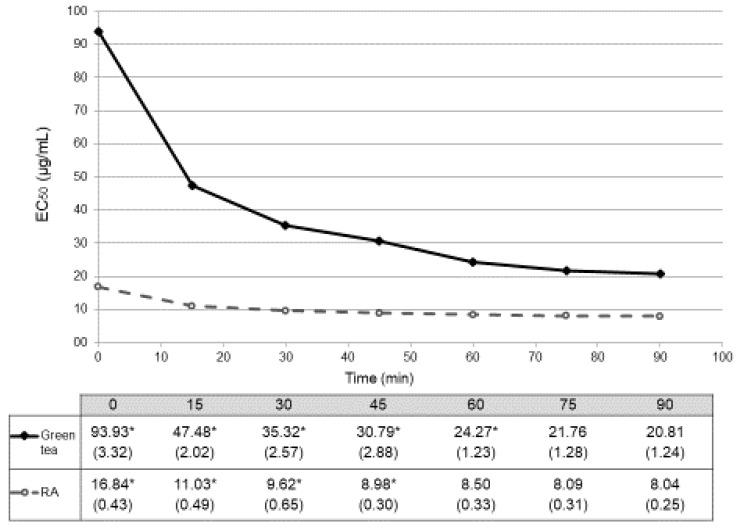
Temporal evolution of antioxidant activity in vivo (DPPH•) expressed in EC_50_ (mean ± SD µg/mL). Dark spots and continuous line correspond to data from treatment with green tea. Empty spots and dashed line correspond to data from treatment with rosmarinic acid (RA). Antioxidant activities of both green tea and RA showed differences in all the time-points (*p* < 0.001). Table below the graph shows the EC_50_ mean values (SD) µg/mL. * indicates the time points in which statistical differences (*p* < 0.05) with their consecutive value could be observed.

As before, reaction of the worm extracts with DPPH• was monitored at 517 nm every 15 min for 90 min, calculating the radical scavenging activity (% inhibition) and EC_50_ values for each concentration at each time point (Table 2). The results obtained for a concentration of 0 µg/mL can also be used as a blank for our method (non-treated worms). Table 2 shows similar radical scavenging activity (% inhibition) within the three groups (at 90 min: 4.51 ± 1.29% for green tea, 5.13 ± 0.29% for rosmarinic acid and 4.67 ± 1.26% for glucose). These values were also represented as a function of time (Figure 3). Again, glucose did not reach 50% of scavenging activity, being in these cases the EC_50_ values higher than 500 µg/mL. In these in vivo conditions, EC_50_ values obtained with treatment with green tea stabilized at 75 min of reaction (EC_50_ = 21.76±1.28 µg/mL, *p* = 0.224 between min 75 and min 90). As before, RA stabilizes earlier in time after 60 min (EC_50_ = 8.50±0.33 µg/mL, *p* = 0.695 between min 60 and min 75) (Figure 3). Antioxidant activities of both green tea and RA were also different at all the time-points (*p* < 0.001).

## 4. Discussion

The free radical theory [1] has led to the search of compounds with an antioxidant activity in order to improve physiological conditions, such as delaying ageing. In general, the starting point is based on the screening of different compounds using in vitro tests like the DPPH• assay. This is the method most frequently used as it is rapid, simple, inexpensive, robust [7] and can be monitored over time, providing an idea of the steady state of the compound. According to [20], this reaction shows three types of kinetics depending on the nature of samples. When the maximum activity is reached before 30 min, the reaction can be catalogued as fast, when it takes more than 30 min to reach the maximum activity, it is intermediate, and when it takes more than 60 min to reach that point, the reaction is slow. In this case, both samples showed different points of stabilization (at 15 min for RA and at 30 min for green tea), so green tea behaves as a compound with an intermediate activity, but RA has a fast activity. As expected, RA has also a higher activity showing a lower EC_50_ value when compared to green tea (2.72 ± 0.11 µg/mL and 1.71 ± 0.04 µg/mL, respectively) [21] as it corresponds to a control component of high activity. Thus, the in vitro behavior can be used as a first screening that can help to consider it as a compound with a potential antioxidant activity before the use of in vivo models. 

However, while in vitro tests are used to demonstrate the intrinsic activity of the compounds, in vivo assays are focused to test the physiological effects, constituting a second and essential line of evidence of antioxidant activity. Unfortunately, sometimes there is no direct relationship between in vitro and in vivo results [22].

In vivo tests are usually performed in complex organisms like mammals, but before doing this it seems more appropriate to perform these assays in simpler organisms, reserving the most complex ones for advanced experimental phases. *C. elegans* is a good organism in which ROS can be manipulated modifying environmental conditions and whose enzymatic antioxidant systems are known [23]. In fact, there are many experiments that have been developed to test antioxidant capacity depending on the pathway of interest. Some assays performed in worms are focused to quantify the expression of specific antioxidant enzymes while others are focused on functional issues like lifespan extension [7,8,24,25]. Most of these assays are also time-consuming (one month approximately for lifespan tests) or need expensive and specialized devices (like fluorescence microscopes in SOD quantification) to be performed [8,9,10]. In addition, they assume that the compound is ingested and directly performs a function since the antioxidant effect is attributed after observing an effect on the worms [24,26]. Thus, antioxidant activity in vivo is indirectly monitored without direct evidence of such activity within the organism, even when the pharyngeal pumping rate is controlled or the intake of the compound is tracked. For example, in vivo methods using *C. elegans* are usually complemented by a quantification of the pharyngeal pumping to ensure the worms are not consuming less [10]. It is not possible to differentiate food source (*E. coli* OP50) from the supplemented compound itself; we simply assume that the worms are eating the compound because there is a pharyngeal movement and the lifespan increases [27], concluding that the compound behaves as an antioxidant in vivo and this is responsible for increasing the lifespan. However, another possibility might be that this was partially an environmental effect.

To quantify total antioxidant capacity in vivo, [28] designed 10 cycles of a shaking and cooling protocol in *C. elegans*. However, we propose a simpler method that eliminates two of its main drawbacks. On the one hand, we propose to crush worms by Ultra-Turrax, avoiding the time-consuming ice-cooling cycles that may not liberate the whole amount of digested compound. On the other hand, the final solution to be tested can be obtained by filtration instead of using centrifugation to separate *C. elegans* cellular debris. This solution can be directly monitored by using the DPPH• method, within the same detection and quantification limits as the in vitro test. The specificity and accuracy of this colorimetric method do not change in vivo as it is still based on the same free radical scavenging activity. To validate this method, we replicated the assay twice from the beginning with two independent stocks of worms. Repeatability was checked performing at least 10 determinations for each compound concentration per experiment replication under the same conditions. On the other hand, our results show coefficient of variation from 2.49 to 9.30% (most of them approximately 4%), being good evidence of reproducibility of the method as they are below 20% [29]. For this reason, our method is precise and valid to quantify in vivo antioxidant activity, reducing the gap between both assays and allowing the direct comparison of the results obtained in both tests.

Our results show that, as expected, RA is more antioxidant than green tea both in vitro and in vivo. Glucose showed a residual antioxidant activity in vitro, although this activity rose up to 26.26 ± 2.70% in the in vivo assay. This could be due to the activation of some antioxidative pathways in the worms [30,31]. *In vivo*, the radical scavenging activity (% inhibition) at 0 µg/mL of concentration of each group (non-treated worms or blank) showed similar and residual values against DPPH•. The kinetics of the reaction of the in vivo assay showed also that RA is a faster antioxidant than green tea, maintaining the time-point differences previously observed in the in vitro test. However, although this approach show evidences in the antioxidant activity of these compounds both in vitro and in vivo, we cannot establish a general conversion factor of the results of both assays [32]. The performance of DPPH• test is the same in both assays (time monitored, DPPH• concentration, volumes, wavelength, and equipment) and results are both expressed in EC_50_ values. A possible difference is that the samples from the in vitro assays were analyzed immediately, so the conditions were optimal for the stability of the compound, while the samples from the in vivo assays were the result of physiological conditions (temperature, presence of digestion enzymes, and pH). For this reason, both EC_50_ values are not completely comparable, although they provide information on the concentration of the compound necessary to produce an internal antioxidant effect in a living organism.

In this sense, a critical point when designing an in vivo experiment points to the concentration of compound to be tested. The lack of knowledge of the therapeutic window of many medicinal plants can lead to choosing concentrations that are too low to exert their effect or too high that they can have toxic effects. In fact, a lack of a functional effect should not always be related to a complete lack of activity [10]. The availability of this simple and rapid method for the screening will help to design experiments in a wide range of concentrations and to compare them with well-known compounds. Generally, results are expressed in terms of radical scavenging activity, but calculating EC_50_ values provides information about the minimum concentration needed to produce a significant effect (at least 50% of the reaction is inhibited) that can be correlated to other tests [33]. For example, in this case, EC_50_ for RA is lower than EC_50_ for the green tea so a higher concentration of green tea will need to produce a similar effect (stock solution of 1.61 mg/mL vs. 4.16 mg/mL, respectively).

Another critical point of the design of these assays is the time of exposure of worms to the compound. Most of in vivo methods using *C. elegans* evaluate this effect during the development of worms from an early larval stage to adult or until they die [8,9,24]. We have performed this assay in the same exposure conditions (after 48 h of treatment of worms in L1 stage); this allows the combination with other assays and comparison with other methods that quantify enzymatic activities or other functional effects.

## 5. Conclusions

In conclusion, the method proposed here is a simple and reproducible assay for *C. elegans* that can be used as a link between in vitro and in vivo experiments. On the one hand, it proves that the compound maintains its antioxidant activity under physiological conditions. On the other hand, it can be used as a first screening assay that guarantees the internal antioxidant activity in *C. elegans* before the use of more complex, expensive, and time-consuming in vivo models.

## Figures and Tables

**Table 1:** Antioxidant activity from the analysis in vitro. Results are expressed as radical scavenging activity (% inhibition) for both green tea and rosmarinic acid (RA). Radical scavenging activity (% inhibition) are mean values ± SD for each concentration (µg/mL) at each time point (min). Inhibition values over 100% are not shown. As shown, glucose (Gluc.) shows values approximately null scavenging activity (0% inhibition).

Sample	Time (min)							
	Concentration (µg/mL)	0	15	30	45	60	75	90
Green tea	500	97.42 ± 0.64	97.45 ± 0.62	97.09 ± 0.57	96.46 ± 0.55	96.17 ± 0.61	95.30 ± 1.06	93.29 ± 1.44
	250	26.54 ± 0.39	96.97 ± 0.42	96.63 ± 0.41	96.29 ± 0.41	95.87 ± 0.50	95.43 ± 0.47	96.64 ± 0.77
	125	96.13 ± 0.70	96.83 ± 0.68	96.52 ± 1.19	95.88 ± 1.18	95.51 ± 0.72	94.98 ± 0.56	94.40 ± 0.99
	62.50	93.71 ± 0.59	95.14 ± 0.51	94.75 ± 0.49	894.01 ± 0.57	93.38 ± 0.44	93.02 ± 0.36	92.44 ± 0.43
	31.25	90.50 ± 1.17	94.50 ± 0.38	94.31 ± 0.17	93.60 ± 0.49	92.82 ± 0.52	92.27 ± 056	91.62 ± 0.61
	15.62	84.57 ± 1.25	93.20 ± 0.47	93.37 ± 0.44	92.86 ± 0.38	92.06 ± 0.51	91.06 ± 0.49	90.43 ± 0.53
	7.81	71.69 ± 1.91	86.90 ± 1.06	88.26 ± 0.92	88.52 ± 1.02	88.27 ± 0.89	88.13 ± 0.81	87.84 ± 0.87
	3.91	44.40 ± 0.40	59.48 ± 1.27	62.64 ± 1.32	63.88 ± 1.51	64.43 ± 1.47	64.84 ± 1.59	64.82 ± 1.58
	1.95	26.48 ± 0.54	35.60 ± 0.74	36.78 ± 1.28	37.07 ± 0.95	36.69 ± 1.25	36.00 ± 1.52	34.75 ± 1.30
	0.98	16.97 ± 1.01	21.14 ± 0.96	21.08 ± 1.42	20.66 ± 1.37	19.83 ± 1.29	19.07 ± 1.36	17.40 ± 1.53
RA	15.62	100.00 ± 3.69	94.09 ± 1.48	93.31 ± 9.69	93.87 ± 1.46	93.53 ± 1.29	92.97 ± 1.40	92.23 ± 1.63
	7.81	97.80 ± 2.98	91.94 ± 1.67	91.99 ± 1.71	92.12 ± 2.53	91.89 ± 1.33	91.57 ± 2.29	91.06 ± 2.63
	3.91	82.66 ± 3.80	84.59 ± 1.72	86.49 ± 9.72	87.84 ± 1.25	88.78 ± 2.03	89.46 ± 1.68	89.60 ± 1.40
	1.95	44.59 ± 3.37	47.77 ± 1.12	50.05 ± 8.20	52.25 ± 1.25	54.08 ± 1.29	54.81 ± 1.52	54.84 ± 1.60
	0.98	18.88 ± 1.85	18.93 ± 1.30	20.68 ± 1.25	21.63 ± 1.24	22.05 ± 1.40	20.98 ± 1.16	18.51 ± 1.72
Gluc.	500	−0.70 ± 1.68	−0.72 ± 7.52	0.09 ± 7.46	0.80 ± 7.40	0.62 ± 7.52	0.40 ± 6.89	1.97 ± 6.79
	250	1.34 ± 4.64	−1.16 ± 5.39	−1.19 ± 5.84	−1.65 ± 17.41	−2.59 ± 16.64	−3.49 ± 18.20	−3.05 ± 19.15
	125	0.38 ± 6.53	−4.18 ± 1.38	−0.48 ± 2.48	−11.67 ± 2.14	−13.21 ± 2.40	−15.00 ± 2.87	−15.30 ± 2.54
	62.50	−0.67 ± 5.96	1.57 ± 9.11	−6.70 ± 6.51	−8.06 ± 16.67	−9.68 ± 17.28	−11.52 ± 8.37	−12.01 ± 8.67
	31.25	4.51 ± 5.03	−6.62 ± 4.69	−8.18 ± 5.06	−9.49 ± 6.03	−11.57 ± 5.47	−14.00 ± 6.78	−14.62 ± 6.83
	15.62	1.07 ± 6.21	2.52 ± 11.54	1.76 ± 11.97	0.17 ± 12.40	−1.86 ± 12.05	−3.90 ± 13.02	−4.83 ± 13.42
	7.81	2.62 ± 3.85	2.93 ± 4.57	1.49 ± 5.18	−0.10 ± 5.91	−2.10 ± 5.26	−4.11 ± 6.49	−4.91 ± 6.85
	3.91	−4.68 ± 7.57	−2.43 ± 1.19	−2.27 ± 1.30	−2.40 ± 2.86	−2.16 ± 2.00	−2.98 ± 2.75	−3.31 ± 2.26
	1.95	−1.09 ± 5.32	−6.39 ± 6.30	−7.42 ± 7.11	−8.72 ± 8.09	−10.88 ± 7.54	−13.26 ± 8.75	−14.03 ± 9.23
	0.98	−2.13 ± 7.00	−7.46 ± 7.58	−8.10 ± 7.98	−9.30 ± 8.53	−11.00 ± 8.53	−12.76 ± 9.08	−12.98 ± 8.82

**Table 2:** Antioxidant activity from the analysis in vivo. Results are expressed as radical scavenging activity (% inhibition) for both green tea, rosmarinic acid (RA), and glucose. Radical scavenging activity (% inhibition) are mean values ± SD. % for each concentration (µg/mL) at each time point (min).

Sample	Time (min)							
	Concentration (µg/mL)	0	15	30	45	60	75	90
Green tea	250	86.25 ± 0.50	87.24 ± 0.76	87.80 ± 0.41	88.42 ± 0.47	88.88 ± 0.66	89.07 ± 0.41	89.89 ± 0.52
	100	55.91 ± 0.90	75.27 ± 0.86	79.91 ± 1.10	82.98 ± 1.52	86.10 ± 1.43	87.17 ± 1.74	88.19 ± 1.45
	50	27.46 ± 0.95	47.49 ± 0.87	54.37 ± 1.58	57.77 ± 1.73	61.84 ± 0.94	63.74 ± 1.10	66.78 ± 1.11
	5	16.55 ± 0.95	17.17 ± 0.95	23.11 ± 0.85	26.31 ± 0.81	28.20 ± 0.89	30.34 ± 1.10	32.54 ± 0.77
	0.5	11.99 ± 0.83	13.74 ± 0.83	18.41 ± 0.91	21.34 ± 1.41	24.78 ± 0.55	27.47 ± 0.75	29.06 ± 0.74
	0	1.32 ± 1.10	2.13 ± 1.19	2.35 ± 1.42	3.18 ± 1.32	3.53 ± 1.27	4.04 ± 1.23	4.51 ± 1.24
RA	250	87.29 ± 0.94	87.72 ± 0.53	88.32 ± 0.57	88.92 ± 0.65	89.37 ± 0.73	89.59 ± 0.88	89.72 ± 0.56
	100	85.60 ± 0.87	86.13 ± 0.65	86.12 ± 0.62	86.31 ± 0.85	87.11 ± 0.83	87.18 ± 0.71	87.24 ± 0.76
	50	83.65 ± 4.24	85.72 ± 0.38	86.27 ± 0.33	86.38 ± 0.33	86.56 ± 0.40	86.66 ± 0.50	86.96 ± 0.42
	5	14.88 ± 0.60	31.30 ± 0.83	34.47 ± 0.80	37.02 ± 0.60	38.71 ± 0.45	38.70 ± 0.73	38.98 ± 0.65
	0.5	4.23 ± 0.52	18.79 ± 0.62	21.53 ± 0.51	23.09 ± 0.55	25.08 ± 1.11	25.10 ± 0.51	25.17 ± 0.54
	0	1.93 ± 0.86	2.53 ± 0.86	2.64 ± 0.89	2.95 ± 1.00	3.51 ± 0.74	4.15 ± 0.83	5.10 ± 0.82
Glucose	250	12.15 ± 0.74	21.73 ± 0.66	22.98 ± 0.83	23.41 ± 0.95	24.03 ± 1.17	25.42 ± 1.43	26.15 ± 1.79
	100	9.47 ± 0.80	10.67 ± 2.82	11.73 ± 2.49	11.95 ± 2.04	13.41 ± 1.89	13.54 ± 1.75	14.92 ± 1.51
	50	6.05 ± 0.47	8.30 ± 0.80	10.29 ± 0.82	11.08 ± 0.88	11.28 ± 0.96	12.20 ± 1.21	13.16 ± 1.19
	5	4.49 ± 0.79	6.95 ± 0.89	7.13 ± 0.82	7.44 ± 0.62	7.26 ± 0.45	8.38 ± 0.58	9.41 ± 0.62
	0.5	3.66 ± 1.00	4.36 ± 0.86	5.38 ± 0.87	5.28 ± 0.81	5.60 ± 0.82	6.49 ± 0.79	6.62 ± 0.93
	0	1.76 ± 0.95	2.07 ± 0.97	2.85 ± 0.68	3.06 ± 1.30	3.40 ± 1.24	3.83 ± 1.30	4.67 ± 1.26
